# Pharmacological Inhibition of Mitochondrial Division Attenuates Simulated High‐Altitude Exposure‐Induced Memory Impairment in Mice: Involvement of Inhibition of Microglia‐Mediated Synapse Elimination

**DOI:** 10.1111/cns.70473

**Published:** 2025-06-08

**Authors:** Panpan Chang, Mengqing Xu, Jiawei Zhu, Jiangpei Bian, Yapeng Lu, Qianqian Luo, Dan Wang, Li Zhu

**Affiliations:** ^1^ Institute of Special Environmental Medicine, Co‐Innovation Center of Neuroregeneration Nantong University Nantong China

**Keywords:** high‐altitude exposure, Mdivi‐1, memory impairment, microglia, phagocytosis of synapses

## Abstract

**Aim:**

To examine the protective effect of mitochondrial division inhibitor‐1 (Mdivi‐1) against high‐altitude‐induced memory impairment in mice.

**Methods:**

C57BL/6J male mice were administered Mdivi‐1 before exposure to a simulated high‐altitude hypoxia environment. The novel object recognition test and Morris water maze were used to test cognitive function. Golgi staining was used to visualize dendritic spines. PCR, Western blot, and immunofluorescence were performed to detect microglial activation and synaptic phagocytosis.

**Results:**

Mice exposed to short‐term or long‐term simulated high‐altitude conditions experienced memory deficits. However, these deficits were significantly mitigated by pre‐treatment with Mdivi‐1. Simulated high‐altitude exposure caused a reduction in synapses (dendritic spines) and the activation of microglia. Following Mdivi‐1 injection, synapse density was significantly increased, and microglial activation was attenuated. Under hypoxic conditions, primary cultured microglia exhibited significantly enhanced phagocytic activity towards TRITC‐Dextran or synaptosomes, which was abolished by Mdivi‐1. Additionally, Mdivi‐1 inhibited the HIF‐1 signaling pathway and restricted the hypoxia‐induced glycolytic activity in microglia. Specific inhibition of glycolysis effectively weakened the phagocytic capacity of microglia under hypoxia.

**Conclusion:**

Mdivi‐1 dramatically mitigated memory impairment in mice induced by simulated high‐altitude exposure. Mdivi‐1 reduced microglial glycolysis in hypoxic conditions, thereby limiting microglial activation and preventing excessive synaptic phagocytosis. Consequently, it effectively protected memory.

## Graphical abstract

The metabolic reprogramming of microglia towards glycolysis under hypoxia promoted their activation and phagocytosis of synapses (dendritic spines), leading to memory loss. Mdivi‐1 inhibits HIF‐1 signaling and glycolytic activity in microglia. Thus, Mdivi‐1 mitigates high‐altitude‐induced spatial memory loss.

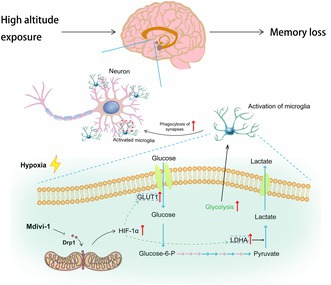

## 1 Introduction

Each year, a significant number of individuals are exposed to high‐altitude environments for various reasons, including work, tourism, and military actions. Recent studies have found that even short‐term exposures to high‐altitude environments can result in memory dysfunction in healthy individuals, as evidenced by impaired maintenance and retrieval of memories acquired before high‐altitude exposure [1, 2]. The deleterious effects of memory deficits, including reduced reaction speed and heightened error rates in decision‐making, can significantly compromise the health and occupational productivity of individuals. Therefore, elucidating the mechanisms underlying high‐altitude exposure‐induced memory impairment and implementing effective protective measures is crucial for improving individuals' health and work efficiency in high‐altitude environments.

In the central nervous system, the encoding and storage of memory require orderly changes in the number, structure, and function of synapses [3]. Dendritic spines are tiny protrusions located on dendritic branches of neurons and are the primary postsynaptic structural basis of excitatory synapses [4]. The changes in the number, size, and shape of dendritic spines are closely related to information storage and retrieval [5]. New dendritic spines are formed during learning and provide the structural basis for memory acquisition, while stable dendritic spines are responsible for memory storage [6]. Clearance of dendritic spines/synapses can lead to forgetting [7, 8]. Research suggests that the obstruction of new dendritic spine generation induced by high‐altitude hypoxia is closely related to cognitive impairment [9, 10]. In a recent study, we found that simulated high‐altitude exposure up‐regulated phagocytosis of microglia, which increased phagocytosis of synapses (dendritic spines) in the hippocampal CA1 region and induced memory loss in mice [11].

It is reported that microglia regulate synaptic density and connectivity, memory strength, and memory quality by making activity‐dependent contact with the synapses [12]. Microglia play an important role in the clearance of dendritic spines/synapses [13]. In the adult brain, activated microglia engulf dendritic spines through phagocytosis, leading to their clearance and influencing the number of dendritic spines and playing a key regulatory role in forgetting [8, 14]. Although high‐altitude exposure has been shown to induce activation of microglia in the brain, the underlying mechanisms are not clear [11, 15].

Mitochondrial dynamics, including mitochondrial fusion and fission, play a crucial role in maintaining the integrity of the mitochondrial network and cellular energy supply [16]. Several studies have found that hypoxia can promote mitochondrial fission by activating Drp1 [17, 18]. Our recent findings suggest that mitochondrial fission may play an important role in microglia activation under hypoxia [15]. It is worth further exploration to investigate whether inhibiting mitochondrial fission under hypoxic conditions can prevent excessive microglia activation, thus reducing excessive phagocytosis of synapses.

Therefore, this study investigated the impact of the mitochondrial fission inhibitor Mdivi‐1 on memory impairment induced by simulated high‐altitude exposure in mice. The effect of glycolysis regulated by mitochondrial fission on microglia‐mediated clearance of dendritic spines/synapses has also been explored.

## 2 Materials and Methods

### 2.1 Materials and Reagents

Mdivi‐1 (SC8028) and 2‐DG (ST1024) were purchased from Beyotime Institute of Biotechnology (Shanghai, CHN). The Golgi staining kit was obtained from HitoBiotec Corp. (HTKNS1125NH, TN, USA). Anti‐IL‐1β (ab9722), anti‐HIF‐1α (ab1), anti‐Iba1(ab5076), and anti‐CD86 (ab239075) antibodies were obtained from Abcam (MA, USA). Anti‐LAMP1 (sc‐19992) antibody was purchased from SANTA Cruz (CA, USA). Anti‐Synaptophysin antibody (101011) was obtained from Synaptic Systems (Goettingen, Germany). Anti‐PSD‐95 (3450T) antibodies were obtained from Cell Signaling Technology (Beverly, USA). Anti‐β‐actin (66009‐1‐lg) and anti‐GLUT1 (21829‐1‐AP) were purchased from Proteintech Group (Wuhan, CHN). Anti‐TREM2 (AF1729) was purchased from R&D Systems. DAPI (D1306) was purchased from Molecular Probes Inc. (Oregon, USA). DMEM/F12 medium, DMEM medium, GlutaMAX, NeuroBasal medium, N2 supplement, and FBS were obtained from Gibco (Carlsbad, USA).

### 2.2 Animals and Treatment

C57BL/6J male mice (SPF grade, 20–25 g) were obtained from the Experimental Animal Center of Nantong University (Nantong, CHN). All mice are housed on a 12‐h light/dark cycle and have free access to food and water. All the studies were submitted to the ethics committee on animal experimentation at Nantong University, and all procedures were approved according to the Animal Care and Use Committee of Nantong University and the Jiangsu Province Animal Care Ethics Committee (Approval ID: SYXK(SU)2007‐0021).

The mice are exposed to an animal decompression chamber (TOW‐INT TECH, Shanghai, CHN), simulating a high altitude of 7000 m for 48 h (HH). The mice were randomly divided into three groups: the control group (Nor) which was exposed to a normoxic condition; the simulated high‐altitude exposure group (HH) which was exposed to a simulated high‐altitude condition; and the Mdivi‐1‐treated group (HH + Mdivi‐1), where mice were pre‐treated with Mdivi‐1 (20 mg/kg, *ip*) once per day for 3 days and then were exposed to HH conditions.

Mice were euthanized via CO_2_ asphyxiation. They were exposed to CO_2_ at a flow rate of 30% of the chamber volume per minute until unconsciousness (loss of righting reflex) was confirmed. The mice were left in the chamber for an additional 3 min to ensure irreversible euthanasia.

### 2.3 Cell Culture

Primary microglia were prepared from the cerebral cortex of newborn mice as previously described [15, 19]. Microglia floating on top of the astrocyte layer were harvested at 12 DIV by shaking at 200 rpm for 2 h and incubated in DMEM/F12 plus GlutaMAX supplemented with 10% FBS and 5 ng/mL GM‐CSF. Iba1 immunofluorescence labeling confirmed that more than 95% of the cells were microglia.

BV2 and HT‐22 cells were obtained from the cell bank of the Chinese Academy of Sciences (Shanghai, CHN). Cells were cultured in the high‐glucose DMEM medium containing 10% FBS and maintained at 37°C in a humidified 5% CO_2_ incubator (Thermo Forma Electron Co., Marietta, OH, USA). HT‐22 were differentiated in NeuroBasal medium containing GlutaMAX and N2 supplement for 24 h before use.

### 2.4 Novel Object Recognition Test

The novel object recognition (NOR) test was conducted in a white, opaque, cubic box measuring 50 cm × 50 cm × 50 cm [20]. The experiment consisted of two main phases. In the first phase (familiarization, T1), two identical objects (A1 and A2) were placed in opposite corners of the box, roughly 10 cm away from the walls. In the second phase (recognition, T2), one of these objects was replaced by a novel one (A = familiar, B = novel).

Each mouse was introduced into the box facing one of the sidewalls and allowed to explore freely for 10 min. After an hour interval, the mice entered the second phase, where they were given another 5 min to explore. During this time, their behavior toward both familiar and novel objects was recorded using behavioral software.

It's important to note that climbing or sitting on an object was not considered exploratory behavior. Instead, sniffing at the object with the nose or touching it with the forepaws was classified as exploration. The discrimination index (DI) was then calculated based on the exploration time (E) of the two objects during T2, using the formula: DI = (EB − EA)/(EA + EB).

### 2.5 Morris Water Maze Test

Morris water maze (MWM) is commonly used to study learning and memory in mice [21]. In this study, we first trained mice to acquire spatial memory by MWM as before [22]. The mice were then treated with simulated high‐altitude exposure and assessed for loss of spatial memory using MWM. On the first day of training, the mice were gently placed in the pool and left free to find a platform to adapt to the experimental environment. The next day, the platform was submerged 1 cm below the surface, and the mice were gently placed into the pool and allowed to find the platform. The same procedure was repeated on subsequent days to train the mice and enable them to acquire spatial memory for the platform. On day eight, the platform was removed and the memory of the mice for the platform was assessed.

### 2.6 Western Blotting Analysis

The samples were homogenized in a lysis buffer, collected, and quantified. The protein samples were then subjected to SDS‐polyacrylamide gel electrophoresis and transferred to PVDF membranes. Membranes were blocked with 5% no‐fat milk in TBST and incubated with the primary antibodies including IL‐1β (1:1000), β‐actin (1:10000), CD86 (1:500), HIF‐1α (1:500), or GLUT1 (1:500) overnight at 4°C. After washing with TBST, the membranes were incubated with the corresponding secondary antibodies for 1 h at room temperature. The images were visualized using a gel imaging system (Tanon 4100, CHN) and analyzed using ImageJ software.

### 2.7 Quantitative Real‐Time PCR Analysis

Total RNA was extracted using Trizol (Ambion, USA) and reverse‐transcribed by HiScript III 1st Strand cDNA Synthesis Kit (Vazyme, Nanjing, CHN). The AceQ qPCR SYBR Green Master Mix (Vazyme, Nanjing, CHN) performed real‐time PCR. The PCR primer sequences are as follows: *Il1b*, 5′–TGC CAC CTT TTG ACA GTG ATG–3′ (forward), 5′–TGA TGT GCT GCT GCG AGA TT–3′ (reverse); *Cd86*, 5′–ACG TAT TGG AAG GAG ATT ACA GCT–3′ (forward), 5′–TCT GTC AGC GTT ACT ATC CCG C–3′ (reverse); *Tnfa*, 5′–AAG CCT GTA GCC CAC GTC GTA–3′ (forward), 5′–GGC ACC ACT AGT TGG TTG TCT TTG–3′ (reverse); *Slc2a1*, 5′–GCT TCT CCA ACT GGA CCT CAA AC–3′ (forward), 5′–ACG AGG AGC ACC GTG AAG ATG A–3′ (reverse); *Ldha*, 5′–ACG CAG ACA AGG AGC AGT GGA A–3′ (forward), 5′–ATG CTC TCA GCC AAG TCT GCC A–3′ (reverse); *β‐actin*, 5′–CAT CCG TAA AGA CCT CTA TGC CAA C–3′ (forward), 5′–ATG GAG CCA CCG ATC CAC A–3′ (reverse). We used the 2^−ΔΔ*Ct*^ method to calculate relative expression.

### 2.8 Immunofluorescence Staining and Assay

The mice were perfused with 4% paraformaldehyde, and the whole brain was harvested and dehydrated in sucrose before embedding in OCT. Coronal sections were cut at 40 μm thickness using a frozen‐stage microtome and stored at −20°C in a slicing protection solution. The brain sections were blocked, washed, and incubated overnight at 4°C with primary antibodies, including synaptophysin (1:800), PSD‐95 (1:400), Iba1 (1:200), LAMP1 (1:1000), or GLUT1 (1:500). Subsequently, brain sections were incubated with fluorescently labeled secondary antibodies. Then, the sections were stained with DAPI. Fluorescence images were taken by using confocal microscopy (SP8, Leica, GER).

Every Iba1‐positive microglia in a particular photomicrograph was analyzed. Consistent with previous methods, we measured the soma area by outlining the cell body with the ImageJ freeform tool, which enabled automatic area calculation [23]. Microglial circularity was assessed using established techniques [11]. Specifically, the fluorescence image was converted to 8‐bit grayscale and optimized for brightness and contrast. We then applied a threshold to distinguish the microglia from the background. The circularity was analyzed using the ImageJ Shape Descriptors tool. Additionally, colocalization analysis was conducted using the ImageJ Colocalization plugin in Coloc 2 Analysis.

### 2.9 Golgi Staining

The experimental procedures were carried out according to the previous descriptions [24]. The mice's brains were placed in a pre‐prepared soaking solution for 24 h and replaced with a fresh soaking solution for 14 days. Coronal sections were cut at 100 μm thickness using a vibrating microtome and the sections were mounted on glass slides coated with 0.03% gelatine and air dried. The sections were then stained using a Golgi staining kit according to the manufacturer's instructions and were observed by a microscope (Leica DMI 4000B, GER).

### 2.10 Brain Tissue Synaptosome Extraction

Fresh brain tissue was homogenized in ice‐cold homogenization buffer and centrifuged at 800 × g for 10 min at 4°C to remove nuclei and cell debris. The supernatant was then collected and centrifuged at 17,000 × g for 20 min at 4°C to pellet the synaptosomes. The pellet was resuspended in a discontinuous sucrose gradient (1.2, 1.0, and 0.8 M sucrose layers) and centrifuged at 82,500 × g for 2 h at 4°C. Synaptosomes were collected from the interface between the 1.0 and 1.2 M sucrose layers, diluted in 10 mM HEPES buffer, and centrifuged at 15,000 × g for 30 min at 4°C. The final pellet was resuspended in DMEM‐F12 and used immediately for co‐incubation with microglia.

### 2.11 Microglia Phagocytosis Assay

We evaluated the microglia phagocytosis using TRITC‐Dextran 40 kDa (AS026, Abclonal) and synaptosomes [11]. The synaptosome mixture was collected and resuspended in a cell medium. The primary microglia were then treated individually for 30 min with either the synaptosome combination or 100 μg/mL TRITC‐Dextran 40 kDa. After that, the cells were fixed and stained using the antibody of synaptosomal markers. A Leica SP8 confocal microscope was used to capture images to measure the fluorescence intensity of TRITC‐Dextran or synaptosomes within the cells.

### 2.12 Cell Viability Assay

Primary microglia or BV2 cells were seeded in a 96‐well plate. Following 24 h of incubation, different concentrations of 2‐deoxy‐D‐glucose (2‐DG) (0, 200, 400, 600, 800, 1000 μM) were added to the medium. The cells were then placed in a hypoxic workstation (Ruskin Technologies, UK) for 24 h under 1% O_2_ conditions. After hypoxic treatment, 10 μL of enhanced CCK‐8 solution (Meilunbio, CHN) was added to each well and incubated for 1 h. The absorbance at 450 nm was measured using a microplate reader (Synergy 2TM, BioTek, Winooski, VT).

### 2.13 Statistical Analysis

All data are presented as mean ± standard deviation (SD). Data were analyzed using GraphPad Prism software. Group comparisons were performed using one‐way or two‐way ANOVA and Student's *t*‐test. Statistical significance was determined as *p* < 0.05.

## 3 Results

### 3.1 Mdivi‐1 Alleviated Memory Impairment in Mice Induced by Simulated High‐Altitude Exposure

**FIGURE 1:**
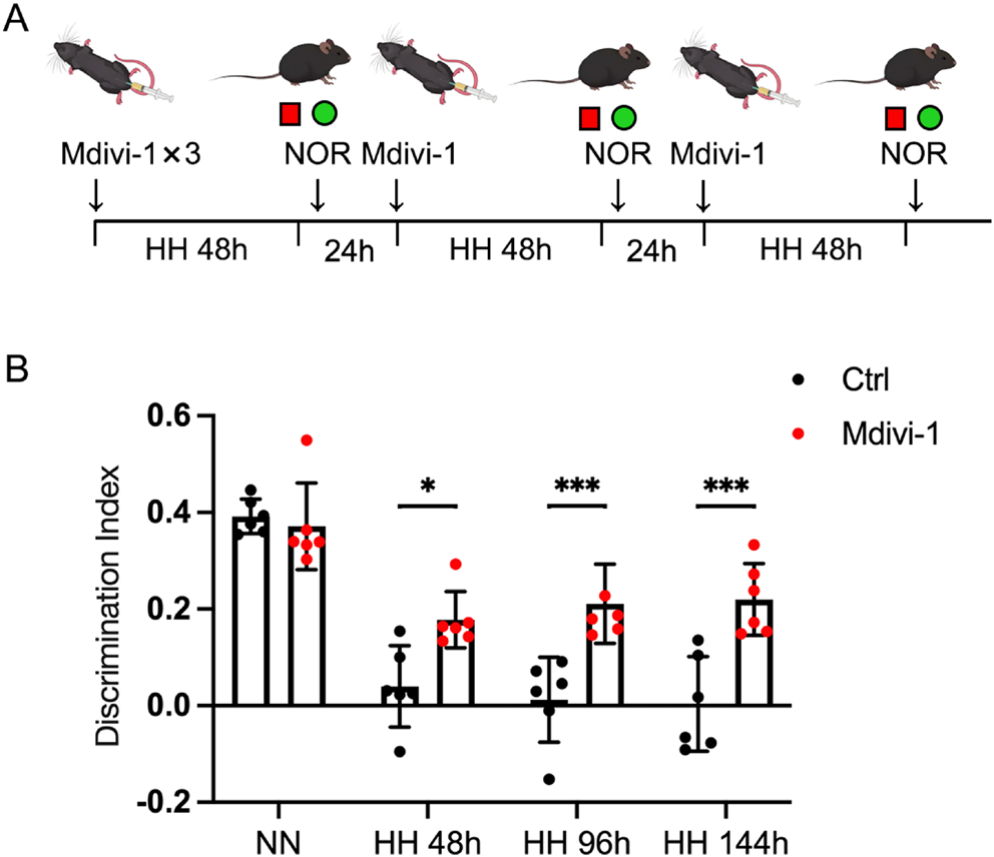
Mdivi‐1 mitigated memory deficits in mice subjected to simulated high‐altitude conditions. (A) C57BL/6 mice were pre‐treated with Mdivi‐1 for 3 days and then exposed to HH conditions. Mdivi‐1 was administered *ip* 30 min after NOR trial. (B) Values of the discrimination index were expressed as means ± SD, *n* = 6. *p < 0.05, ***p < 0.001.

We first constructed the model as shown in Figure 1A. The NOR test was employed to assess the DI of mice following varying durations of simulated high‐altitude exposure. Our findings indicated that simulated high‐altitude exposure caused a significant decline in the DI of the mice. However, administration of Mdivi‐1 resulted in a notable recovery of the DI (Figure 1B). This change was observed not only after short‐term hypoxic exposure lasting 48 h but also under conditions of long‐term, intermittent simulated high‐altitude exposure. When mice were exposed to high‐altitude conditions, their DI value decreased. The results suggested that these mice may be unable to effectively distinguish between novel and familiar objects, indicating potential memory impairment or forgetfulness. However, Mdivi‐1 can effectively reverse DI decline.

**FIGURE 2:**
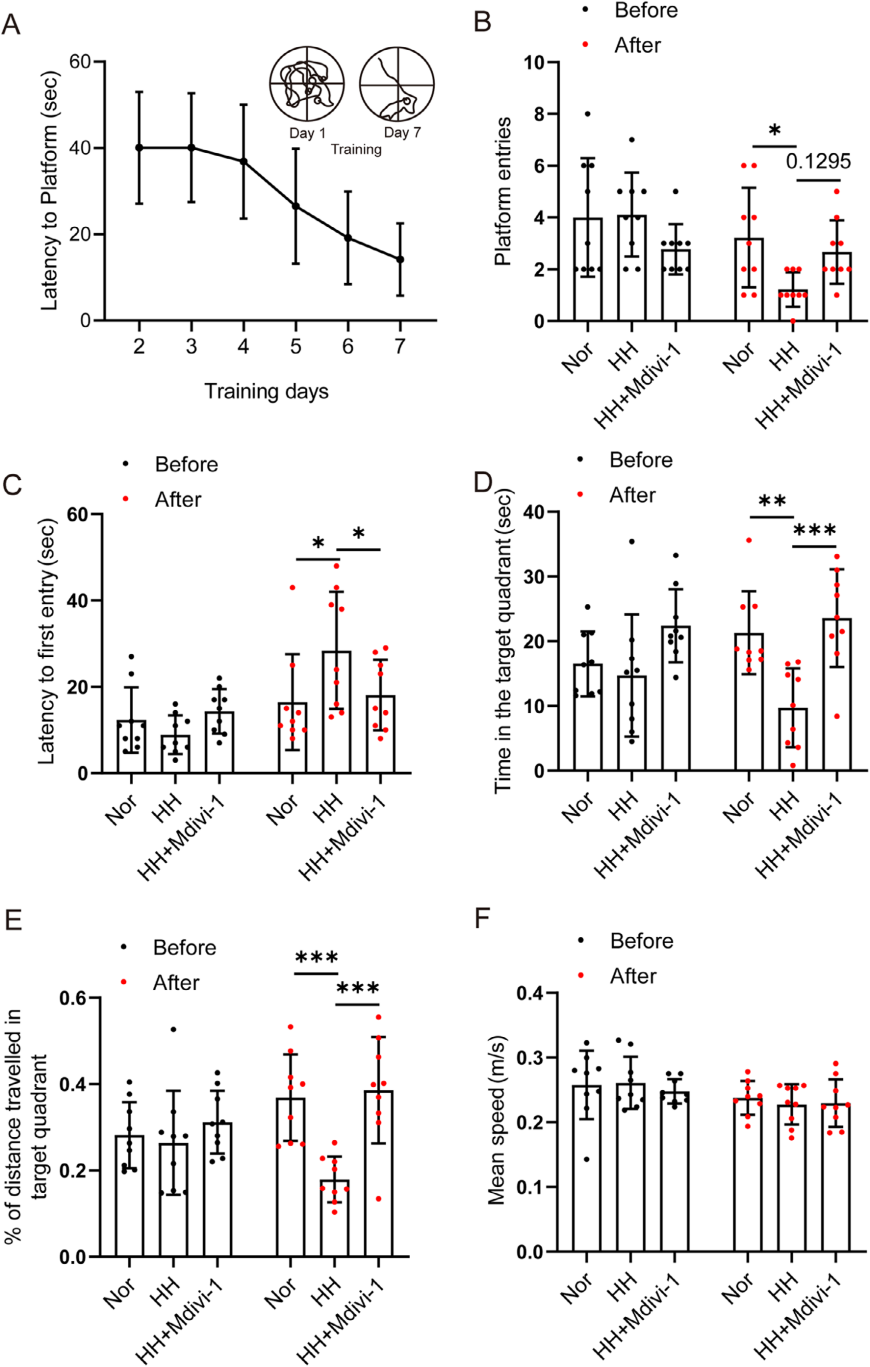
Mdivi‐1 alleviated spatial memory loss in mice induced by simulated high‐altitude exposure. C57BL/6 mice were trained by MWM and exposed to a simulated altitude of 7000 m for 48 h. (A) The latency to the platform from day 2 to day 7 during MWM training. (B) The platform entries, (C) the latency to the first entry, (D) the time in the target quadrant, (E) the distance traveled in the target quadrant, and (F) the mean speed of mice before or after a simulated high‐altitude exposure are recorded by MWM. Before: before simulated high‐altitude exposure treatment; After: after simulated high‐altitude exposure treatment. Data are expressed as means ± SD, *n* = 9. *p < 0.05, **p < 0.01, ***p < 0.001.

Subsequently, we utilized the MWM to evaluate the impact of 48‐h simulated high‐altitude hypoxia on the memory performance of mice. After 6 days of MWM training, C57BL/6J mice acquired spatial memory of the platform because the time it took for the mice to first enter the platform was gradually decreasing (Figure 2A). Then, the spatial memory of the mice was evaluated using the MWM test after the corresponding treatments. The results showed that the number of entries into the platform, the time spent, and the percentage of distance traveled in the quadrant where the target platform was located were all lower in the HH group than in the Nor group. Additionally, the time it took for the HH mice to first enter the platform was longer than that of the Nor group, indicating that simulated high‐altitude exposure induced forgetting of spatial memory. However, pretreatment with the mitochondrial fission inhibitor Mdivi‐1 alleviated the spatial memory loss induced by simulated high‐altitude exposure, as evidenced by a significant increase in the number of times the mice entered the platform, the time spent, and the percentage of distance in the target platform quadrant compared to the HH group. Besides, the time to first enter the platform was shorter in the Mdivi‐1 group than in the HH group (Figure 2B–E). In the MWM test, the mean speed of the mice remained unchanged, indicating that simulated high‐altitude exposure or Mdivi‐1 treatment had no significant effect on locomotor performance (Figure 2F).

### 3.2 Mdivi‐1 Attenuated the Reduction in the Number of Synapses in Mice Brains Induced by Simulated High‐Altitude Exposure

**FIGURE 3:**
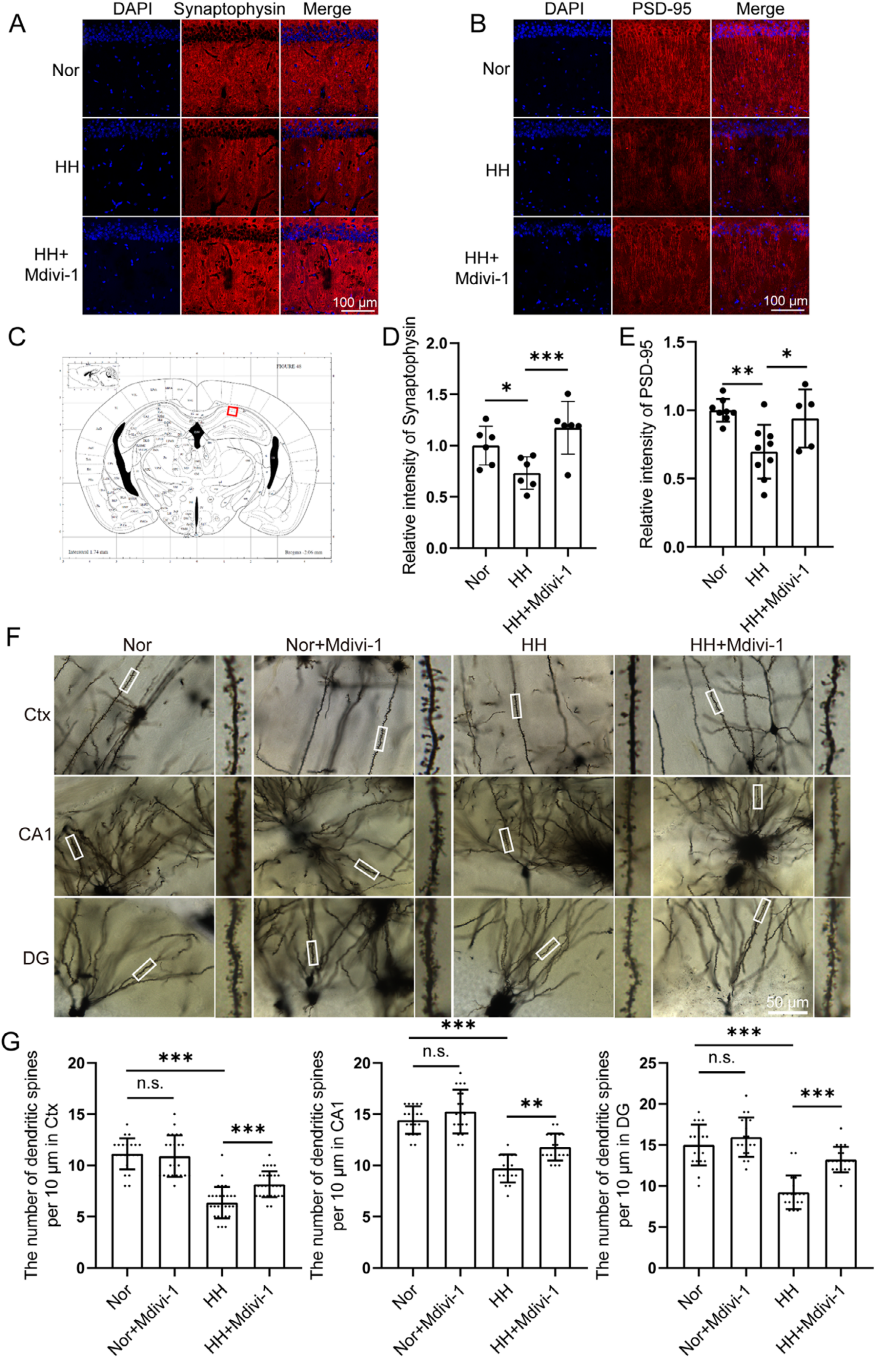
Mdivi‐1 attenuated the reduction in the number of synapses in mice brains induced by simulated high‐altitude exposure. C57BL/6 mice were pretreated with Mdivi‐1 and then exposed to a simulated altitude of 7000 m for 48 h. (A, B) Synaptophysin and PSD‐95 were observed by immunofluorescence, respectively. Scale bar = 100 μm. (C) Whole brain map, marking the location of the hippocampus. (D, E) Quantitative analysis of the intensity of Synaptophysin and PSD‐95 in panels (A, B), respectively. Synaptophysin: *n* = 6. PSD‐95: Nor group *n* = 8, HH group *n* = 9, HH + Mdivi‐1 group *n* = 5. (F, G) The numbers of dendritic spines in the cortex, CA1 and DG regions were observed by Golgi staining. Scale bar = 50 μm. (Ctx: *n* = 23 in Nor and Nor + Mdivi‐1, *n* = 30 in HH and HH + Mdivi‐1. CA1: *n* = 19 in Nor, Nor + Mdivi‐1, and HH + Mdivi‐1, *n* = 17 in HH. DG: *n* = 19. Brain sections from 3 mice of each group were stained, and 5–11 dendrites of each mouse in the indicated region were quantified.). Data are expressed as means ± SD, *p < 0.05, **p < 0.01, ***p < 0.001.

The immunofluorescence intensity results showed that synaptophysin or PSD‐95 in the hippocampal CA1 region of the HH group was significantly lower than that of the Nor group, indicating that the number of synapses in the HH group was lower than that in the Nor group. Pre‐treatment with Mdivi‐1 alleviated the reduction in the number of synapses induced by simulated high‐altitude exposure for 48 h (Figure 3A–E). A similar trend was observed in the outcomes of long‐term, intermittent exposures to simulated high‐altitude hypoxia (Figure S1).

Next, the Golgi staining results showed that the number of dendritic spines in the cortical region, hippocampal CA1 region, and DG region was significantly reduced after simulated high‐altitude exposure, while Mdivi‐1 effectively alleviated the reduction of dendritic spine number induced by high‐altitude exposure (Figure 3F,G).

### 3.3 Mdivi‐1 Attenuated the Phagocytosis of Synapses in Microglia Induced by Simulated High‐Altitude Exposure

**FIGURE 4:**
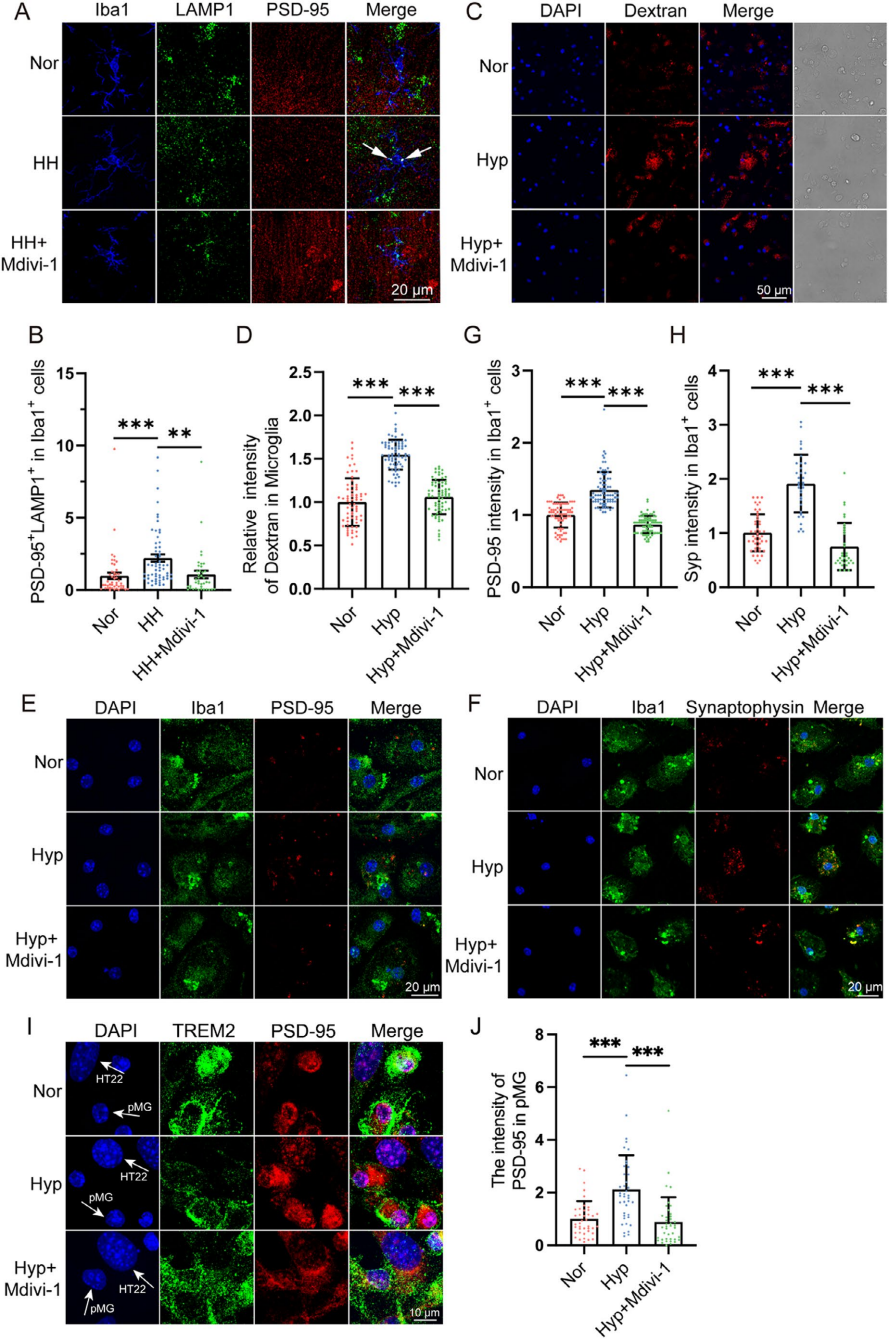
Mdivi‐1 attenuated the phagocytosis of synapses by microglia induced by simulated high‐altitude exposure. (A) C57BL/6 mice were pretreated with Mdivi‐1 and then exposed to a simulated altitude at 7000 m for 48 h. Iba1, LAMP1, and PSD‐95 were labeled by Immunofluorescence. Scale bar = 20 μm. (B) Quantitative analysis of the co‐location of LAMP1 and PSD‐95 in Iba1^+^ cells in panel (A) (*n* = 47 in Nor, *n* = 59 in HH, and *n* = 39 in HH + Mdivi‐1. Brain sections from 6 mice of each group were stained, and 6–10 cells of each mouse were quantified). (C) Primary cultured microglia were pretreated with Mdivi‐1 and exposed to 1% O_2_ for 24 h. Then, the cells were incubated with TRITC‐Dextran 40 kDa for 30 min, and confocal images were recorded. Scale bar = 50 μm. (D) Quantitative analysis of the fluorescence intensity of Dextran in panel (C), *n* = 67. (E, F) Primary cultured microglia were pretreated with Mdivi‐1 and exposed to 1% O_2_ for 24 h, and incubated with dissociated synapses for 30 min. The intensity of PSD‐95 and Synaptophysin in Iba1^+^ cells were detected by immunofluorescence. Scale bar = 20 μm. (G, H) Statistics of the total fluorescence intensity of PSD‐95 in panel (E, *n* = 76 in Nor, *n* = 75 in Hyp, *n* = 78 in Hyp + Mdivi‐1 or Synaptophysin) and panel (F, *n* = 47 in Nor, *n* = 34 in Hyp, *n* = 33 in Hyp + Mdivi‐1) in microglia. (I) HT‐22 cells co‐cultured with primary microglia were treated with 1% O_2_ for 24 h. The PSD‐95 and TREM2 were labeled by Immunofluorescence. (J) Statistics of the total fluorescence intensity of PSD‐95 in TREM2^+^ cells in panel (I), *n* = 40. Scale bar = 10 μm. Data are expressed as means ± SD, **p < 0.01, ***p < 0.001.

Next, we assessed the phagocytic activity of microglia. Co‐localization of LAMP1 and PSD‐95 in microglia served as an indicator of synaptic engulfment. Compared to the Nor group, the HH group exhibited intensity of double‐positive fluorescence signals, suggesting that hypoxia enhanced the phagocytic capacity of microglia. However, pre‐treatment with Mdivi‐1 markedly attenuated the hypoxia‐induced increase in microglial phagocytosis of synapses (Figure 4A,B).

In primary microglia cultured ex vivo, we obtained similar findings. When co‐cultured with TRITC‐Dextran or synaptosomes isolated from the brain, microglia from the Hyp group exhibited a significantly greater uptake of TRITC‐Dextran (Figure 4C,D)or synaptosomes (Figure 4E–H). The hypoxia‐induced enhancement in phagocytic activity was markedly attenuated following pre‐treatment with Mdivi‐1.

To further confirm the direct effect of hypoxia on microglia phagocytosis of synapses, we established a co‐culture system comprising primary microglia and HT‐22. As shown in Figure 4I,J, a significant augmentation in PSD‐95 signal in microglia was observed following hypoxic exposure. Moreover, Mdivi‐1 pre‐treatment mitigated phagocytosis of synapses by microglia.

### 3.4 Mdivi‐1 Inhibited the Activation of Microglia Induced by Simulated High‐Altitude Exposure

**FIGURE 5:**
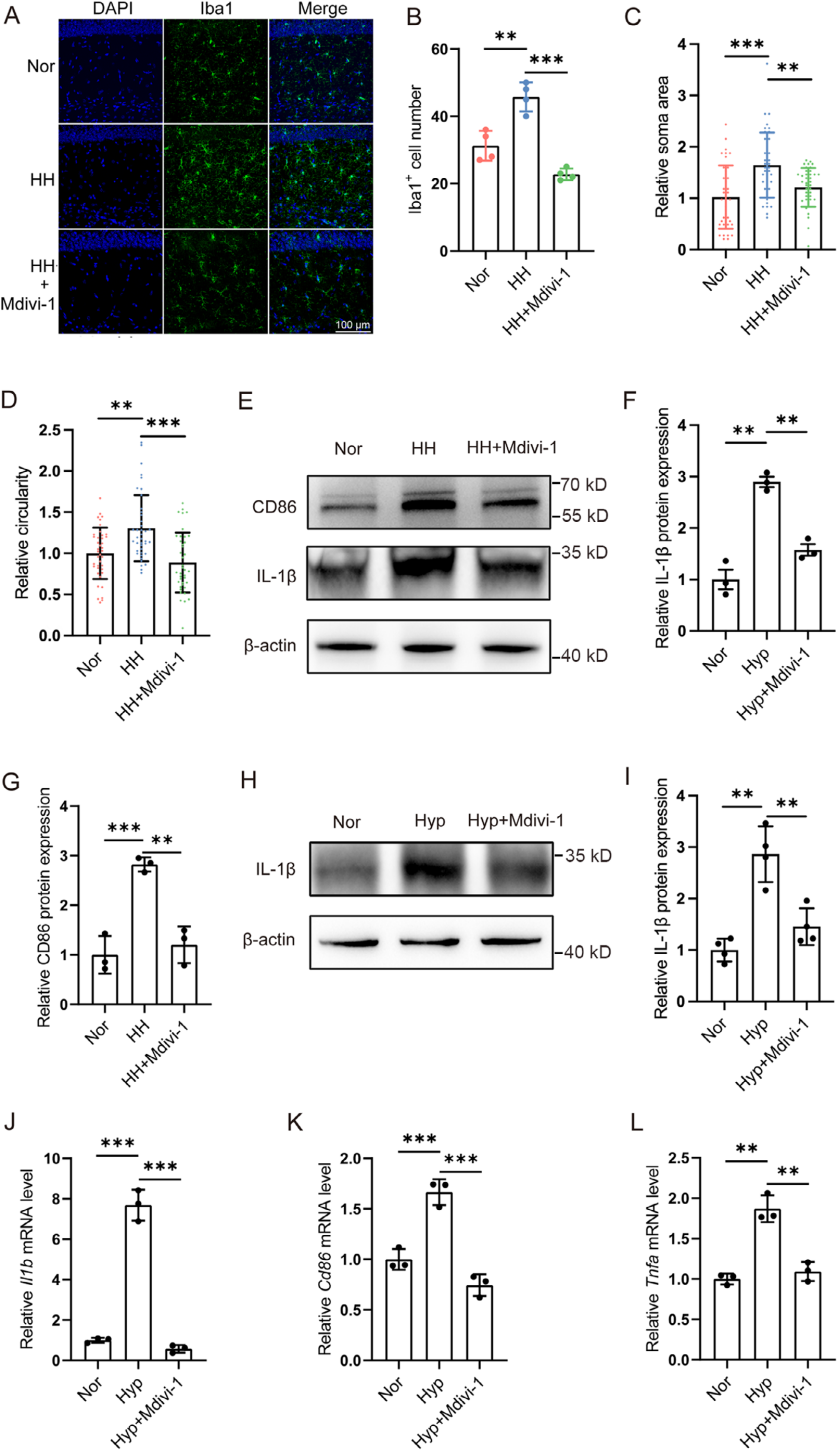
Mdivi‐1 inhibited the activation of microglia induced by simulated high‐altitude exposure. (A) C57BL/6 mice were pretreated with Mdivi‐1 and then exposed to a simulated altitude at 7000 m for 48 h. Immunofluorescence labeling of Iba1 in CA1. Scale bar = 100 μm. (B–D) Statistics of panel (A), including the number of Iba1^+^ cells (B, *n* = 4), soma area (C), circularity (D), *n* = 40. (Brain sections from 4 mice of each group were stained, and 8–12 cells of each mouse were quantified.) (E–G) Mice were pretreated with Mdivi‐1 and then exposed to a simulated altitude at 7000 m for 48 h, the protein level of IL‐1β and CD86 was detected by Western blotting. (H, I) Primary cultured microglia were pretreated with Mdivi‐1 and exposed to 1% O_2_ for 24 h, the protein level of IL‐1β was detected by Western blotting. *n* = 4. (J–L) Primary cultured microglia were pretreated with Mdivi‐1 and then exposed to 1% O_2_ for 24 h, the mRNA levels of *Il1b*, *Cd86*, and *Tnfa* were analyzed by quantitative real‐time PCR and normalized with β‐actin. *n* = 3. Data are expressed as means ± SD, **p < 0.01, ***p < 0.001.

To investigate the effects of HH exposure, we labeled the microglia marker Iba1 in the hippocampal region (Figure 5A–D). The results showed that mice exposed to simulated high altitude increased the number of Iba1‐positive cells, as well as their circularity and soma area. However, microglia activation in the Mdivi‐1 pre‐treated group was significantly inhibited. Western blotting analysis showed that Mdivi‐1 pre‐treatment significantly inhibited the elevation of M1‐type polarization markers IL‐1β and CD86 in microglia induced by simulated high altitude exposure (Figure 5E–G). Mdivi‐1 also inhibited the increase in IL‐1β protein level in microglia induced by hypoxia (Figure 5H,I). After exposure to hypoxia, the mRNA levels of M1‐type polarization markers *Il1b*, *Cd86*, and *Tnfa* in microglia were significantly increased, but Mdivi‐1 pre‐treatment inhibited the elevation of these markers (Figure 5J,K).

### 3.5 Mdivi‐1 Inhibited the Glycolytic Activity in Hypoxia‐Treated Microglia

**FIGURE 6:**
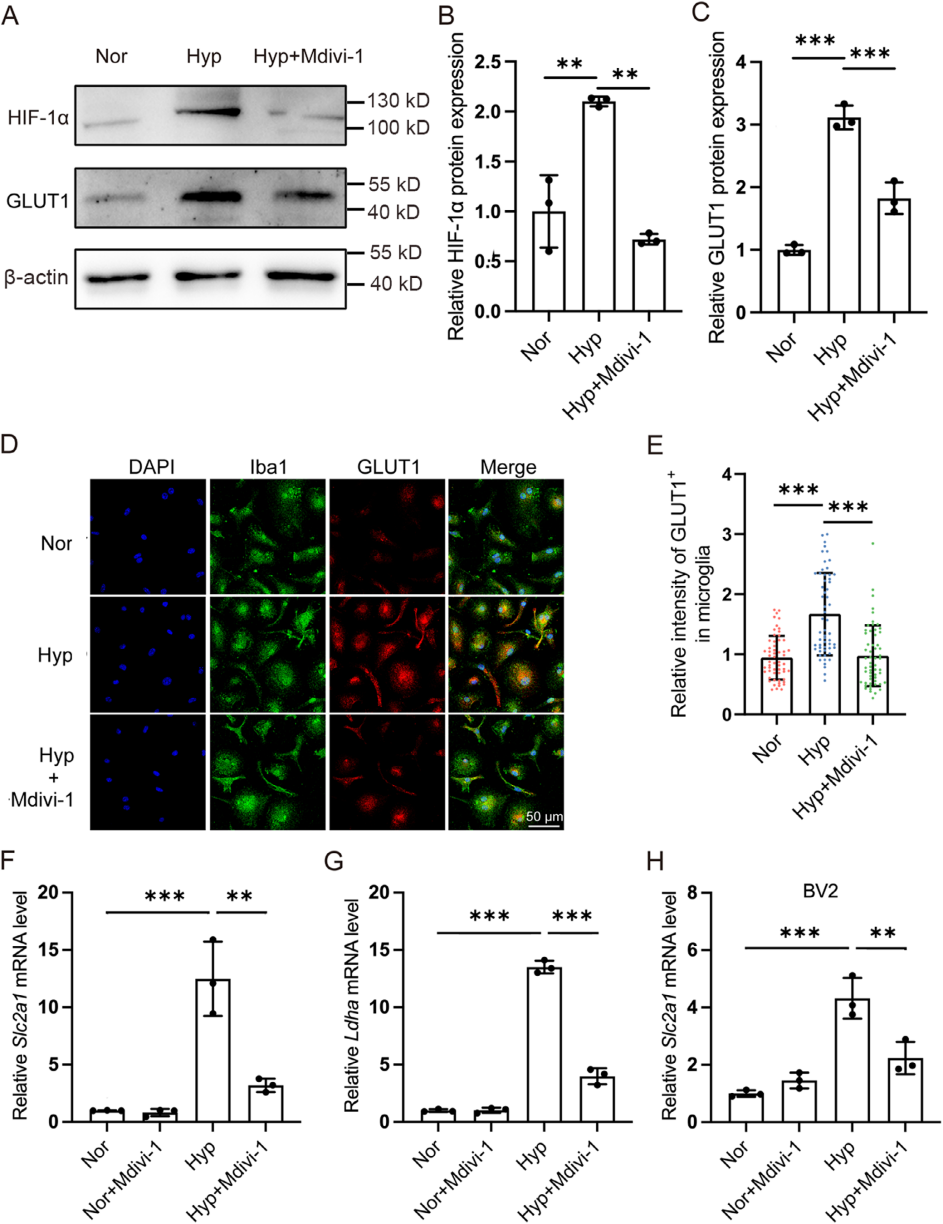
Mdivi‐1 inhibited the increase of glycolytic activity in hypoxia‐treated microglia. Primary cultured microglia were pretreated with Mdivi‐1 and exposed to 1% O_2_ for 24 h. (A–C) The protein levels of HIF‐1α and GLUT1 were detected by Western blotting, *n* = 3. (D) Immunofluorescence labeling of Iba1 and GLUT1, Scale bar = 50 μm. (E) Quantitative analysis of GLUT1 in microglia was described in panel (D), *n* = 57. (F, G) The mRNA level of *Slc2a1* and *Ldha* was analyzed by quantitative real‐time PCR (qRT‐PCR) and normalized with β‐actin. *n* = 3. (H) BV2 cells were pretreated with Mdivi‐1 exposed to hypoxia for 24 h, the mRNA level of *Slc2a1* was analyzed by qRT‐PCR and normalized with β‐actin, *n* = 3. Data are expressed as means ± SD, **p < 0.01, ***p < 0.001.

HIF‐1α is a key factor in regulating glycolysis under hypoxic conditions. It was found that the protein level of HIF‐1α in microglia was significantly increased under hypoxic conditions, while Mdivi‐1 treatment effectively inhibited the enhancement of HIF‐1α protein level induced by hypoxia. Not surprisingly, Mdivi‐1 also significantly reduced the upregulation of the HIF‐1 target gene, glucose transporter 1 (*Slc2a1*) under hypoxic conditions (Figure 6A–E). In addition, Mdivi‐1 pre‐treatment effectively suppressed the hypoxia‐induced upregulation of mRNA levels of *Slc2a1* and *Ldha* (Figure 6F–H).

### 3.6 Inhibition of Glycolytic Activity Attenuated Hypoxia‐Induced Phagocytosis of Microglia

**FIGURE 7:**
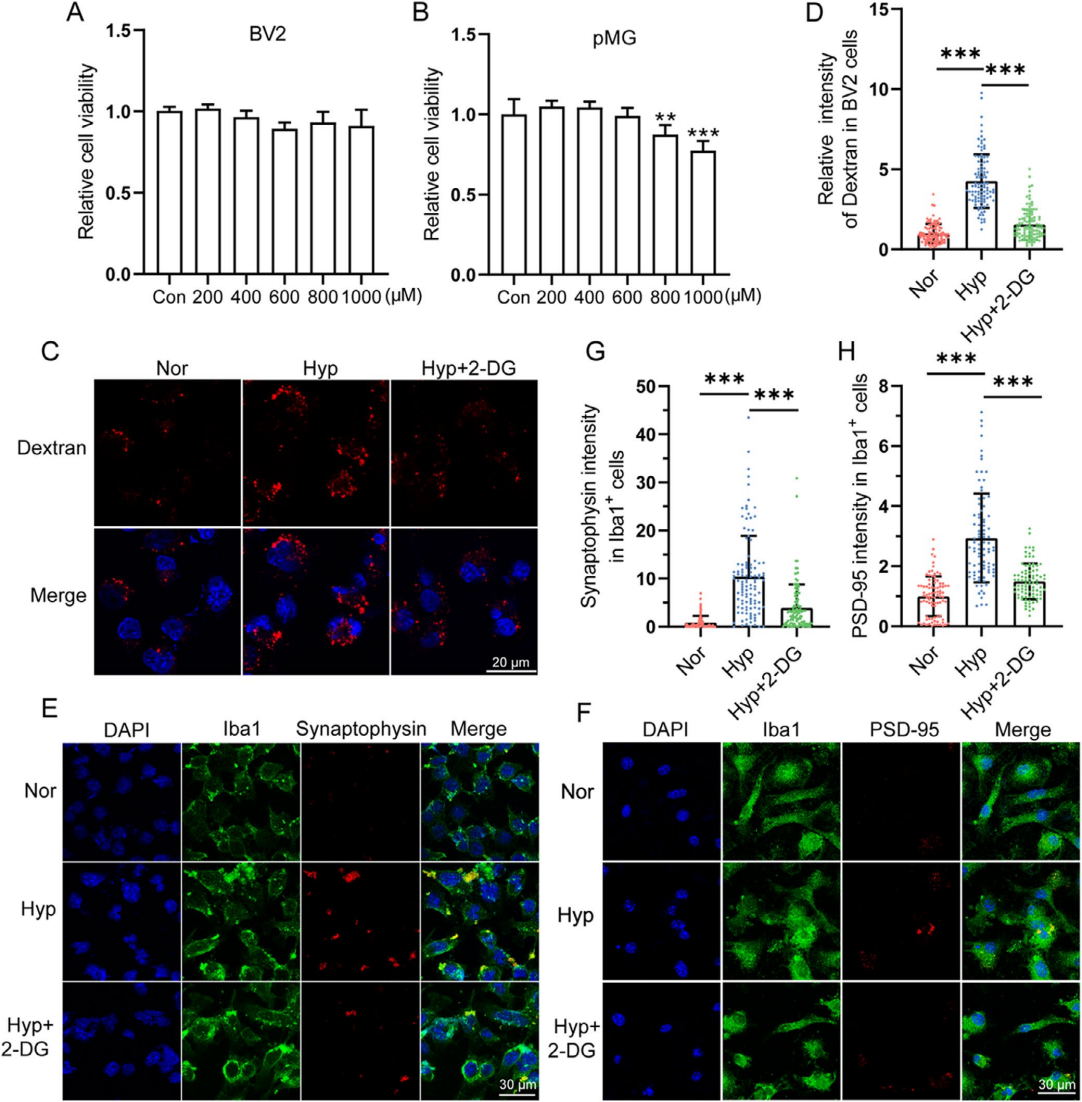
Inhibition of glycolytic activity attenuated hypoxia‐induced phagocytosis of microglia. (A) BV‐2 cells and primary microglia cells were treated with 1% O_2_ and 2‐DG (200–1000 μM) for 24 h, and cell viability was detected by CCK‐8 assay. *n* = 6. (C, D) BV2 cells were treated with 2‐DG and exposed to 1% O_2_ for 24 h. Following a 30‐min incubation period with TRITC‐Dextran 40 kDa, confocal pictures were captured. Scale bar = 20 μm. *n* = 100. (E) BV2 cells and (F) primary cultured microglia were treated with 2‐DG and exposed to 1% O_2_ for 24 h and incubated with dissociated synapses for 30 min. Iba1, Synaptophysin and PSD‐95 were detected by immunofluorescence. Scale bar = 30 μm. (G, H) Quantitative analysis of Synaptophysin or PSD‐95 in Iba1^+^ cells in panel (E, F) (Synaptophysin: *n* = 112; PSD‐95: *n* = 88). Scale bar = 30 μm. Data are expressed as means ± SD, ***p < 0.001.

The glycolysis inhibitor 2‐DG was used to observe the changes in microglial phagocytic ability under hypoxic conditions. Firstly, CCK‐8 results showed that 2‐DG at 1000 μM did not produce significant cytotoxicity to BV2, while 2‐DG at concentrations lower than 600 μM also showed no significant cytotoxicity to primary microglia (Figure 7A,B). Based on these results, subsequent experiments selected 1000 μM of 2‐DG to treat BV2 and 600 μM of 2‐DG to treat primary microglia.

Results in Figure 7C,D showed that 2‐DG effectively inhibited hypoxia‐induced phagocytosis of TRITC‐dextran in BV2. In addition, we examined the effect of 2‐DG on the ability of BV2 or primary microglia to phagocytose synaptosomes isolated from mouse brains. The fluorescence signal of synaptosomes in BV2 or primary microglia in the Hyp group was significantly higher than that in the Nor group, while the fluorescence signal in the 2‐DG + Hyp group was significantly lower than that in the Hyp group (Figure 7E–H). These findings indicated that inhibition of glycolytic activity significantly reduced the hypoxia‐induced enhancement of synaptosome phagocytosis.

## 4 Discussion

It is now widely accepted that the impairment of learning and memory function in high‐altitude environments is altitude‐dependent [25, 26]. Most studies have focused on the effects of the high‐altitude environment on learning processes and memory formation, with less attention being paid to memory maintenance and extraction [11]. Our study found that both short‐term and intermittent long‐term simulated high‐altitude hypoxic exposures significantly impair memory in mice. The NOR test showed that mice exposed to high‐altitude conditions decreased object recognition ability, which can be interpreted as forgetting familiar objects. Additionally, mice trained to form spatial memory by MWM significantly forgot their platform memories after simulated high‐altitude exposure. In behavioral tests, administration of the mitochondrial division inhibitor Mdivi‐1 significantly attenuated forgetting of familiar objects and spatial memory loss, suggesting its potential application as a memory protector under high‐altitude exposure.

Neurons are interconnected through the formation of synapses, which are the structural basis for information storage and retrieval [3]. The encoding and storage of memories requires changes in the number, structure, and function of synapses, which are known as synaptic plasticity [27]. The dendritic spines, the tiny protruding structures located on the dendritic branches of neurons, are the structural basis for information storage and extraction [5]. High‐altitude exposure‐induced impairment of new dendritic spine production is closely associated with learning disabilities [9, 10]. In the present study, we further confirmed that simulated high‐altitude exposure led to a significant decrease in synapses (dendritic spines) number in the hippocampal region. Mdivi‐1 pre‐treatment attenuated the reduction of synapses (dendritic spines), which may be an important mechanism for the memory‐protective effects under high‐altitude exposure.

Microglia prune excess synapses to optimize neural networks, which is vital for cognitive abilities such as learning and memory. A recent study found that microglia can engulf synaptic structures of hippocampal neurons, potentially destabilizing memories and contributing to forgetting [8, 28]. Microglia engulf and clear dendritic spines upon their activation [29]. Activated microglia exhibited enhanced phagocytic activity towards synaptosomes. Pre‐treatment with Mdivi‐1 significantly reduced microglial activation and phagocytic capacity under simulated high‐altitude exposure. The enhanced phagocytic activity of activated microglia requires a high energy supply [30]. Microglia exhibit metabolic flexibility, reprogramming their mitochondrial function to meet their energy demands [31]. Using peripheral immune cells demonstrates that polarization to an M1 phenotype is often accompanied by a shift from oxidative phosphorylation to aerobic glycolysis for energy production [32, 33]. Glucose uptake in microglia is facilitated predominately by GLUT1, particularly under inflammatory conditions. Blocking GLUT1‐mediated glucose uptake reduced glycolysis and suppressed phagocytosis [34]. We employed 2‐DG to limit the microglia glycolytic activity, and the ability of microglia to engulf synapses was significantly inhibited. Our study confirmed that hypoxia‐induced microglial activation was accompanied by increased glycolytic activity. Although glycolysis generates less ATP than oxidative phosphorylation, glucose metabolism in glycolysis is 10–100 times faster. The rapid production of ATP promotes microglial proliferation, migration, cytokine secretion, and phagocytosis [35]. Mitochondrial morphology is dynamic and sensitive to metabolic alteration [36]. Enhancing mitochondrial fission promotes a glycolytic shift [37, 38]. Our previous study showed that Mdivi‐1 effectively reduced mitochondrial fission by inhibiting Drp1 phosphorylation under simulated high‐altitude exposure [15]. Mdivi‐1 may protect memory by inhibiting microglia glycolytic activity to attenuate the abnormal microglial activation.

Inflammation and oxidative stress are common factors that activate microglia. Some studies have shown that neuroinflammation and oxidative stress were suppressed via NF‐κB and Keap1/Nrf2/HO‐1 pathways following Mdivi‐1 administration [39]. Our data demonstrated that Mdivi‐1 inhibited inflammation, characterized by downregulated expression of pro‐inflammatory cytokines. It suggested that Mdivi‐1 attenuated microglia activation through the suppression of inflammatory responses, thereby exerting a protective effect on memory function. Given the known association between blood–brain barrier (BBB) permeability and cognitive decline [40], our previous study demonstrated that Mdivi‐1 can attenuate BBB damage [15]. Numerous studies have confirmed that Mdivi‐1 can cross the BBB to improve cognitive function [41, 42, 43, 44]. The evidence showed that Mdivi‐1 may also affect cognitive function by maintaining BBB integrity.

Although our study provides valuable insights into the role of Mdivi‐1 in alleviating high‐altitude memory impairment, it also has certain limitations. Using an animal decompression chamber, we can only simulate the high altitude and cannot fully replicate other high‐altitude environmental factors, such as cold and ultraviolet radiation. Although hypoxia is the most prominent key factor influencing brain physiological processes, other unique plateau environmental factors can also have specific effects. In addition, our investigation unveiled that Mdivi‐1 affects the glycolytic process of microglia, but the molecular mechanisms underlying it still require further investigation.

## 5 Conclusion

The simulated high‐altitude exposure induces memory impairment in mice. Our study suggests that the metabolic reprogramming of microglia towards glycolysis under hypoxia promotes their activation and phagocytosis of synapses (dendritic spines), leading to memory deficiency. Mdivi‐1, a mitochondrial division inhibitor, suppresses the hypoxia‐induced microglial glycolytic activity and reduces microglial phagocytosis of synapses (dendritic spines), allowing for memory retention. Mdivi‐1 is a potential memory protector under high‐altitude exposure.

## Author Contributions

Panpan Chang: investigation, methodology, formal analysis, and writing – original draft. Mengqing Xu: investigation, methodology, formal analysis, and writing – original draft. Jiawei Zhu: validation and methodology. Jiangpei Bian: investigation. Yapeng Lu: validation, writing – original draft. Qianqian Luo: validation and funding acquisition. Dan Wang: conceptualization, resources, writing – review and editing, supervision, and project administration. Li Zhu: resource, supervision, project administration, funding acquisition.

## Ethics Statement

All the studies reported here were submitted to the ethics committee on animal experimentation at Nantong University, and all procedures were approved according to the Animal Care and Use Committee of Nantong University and the Jiangsu Province Animal Care Ethics Committee (Approval ID: SYXK(SU)2007–0021).

## Conflicts of Interest

The authors declare no conflicts of interest.

## Supporting information


**Figure S1.** Mdivi‐1 attenuated the reduction in the number of synapses in mice brains induced by long‐term, intermittent simulated high‐altitude exposure. Mice were pretreated with Mdivi‐1 and then exposed to HH 3 times as described in Figure 1A. (A, B) The levels of PSD‐95 and Synaptophysin were observed by immunofluorescence, respectively. Scale bar = 100 μm. (C, D) Quantitative analysis of the intensity of PSD‐95 and Synaptophysin was performed in panels (A, B), respectively. Data were expressed as means ± SD (*n* = 10). ***p < 0.001.

## Data Availability

The data supporting this study's findings are available on request from the corresponding authors.
