# Supplementary figures and images for: Pharmacological Inhibition of Mitochondrial Division Attenuates Simulated High‐Altitude Exposure‐Induced Memory Impairment in Mice: Involvement of Inhibition of Microglia‐Mediated Synapse Elimination
Source: CNS Neurosci Ther. 2025 Jun 8;31(6):e70473. doi: 10.1111/cns.70473 (PMC12146113; doi:10.1111/cns.70473)

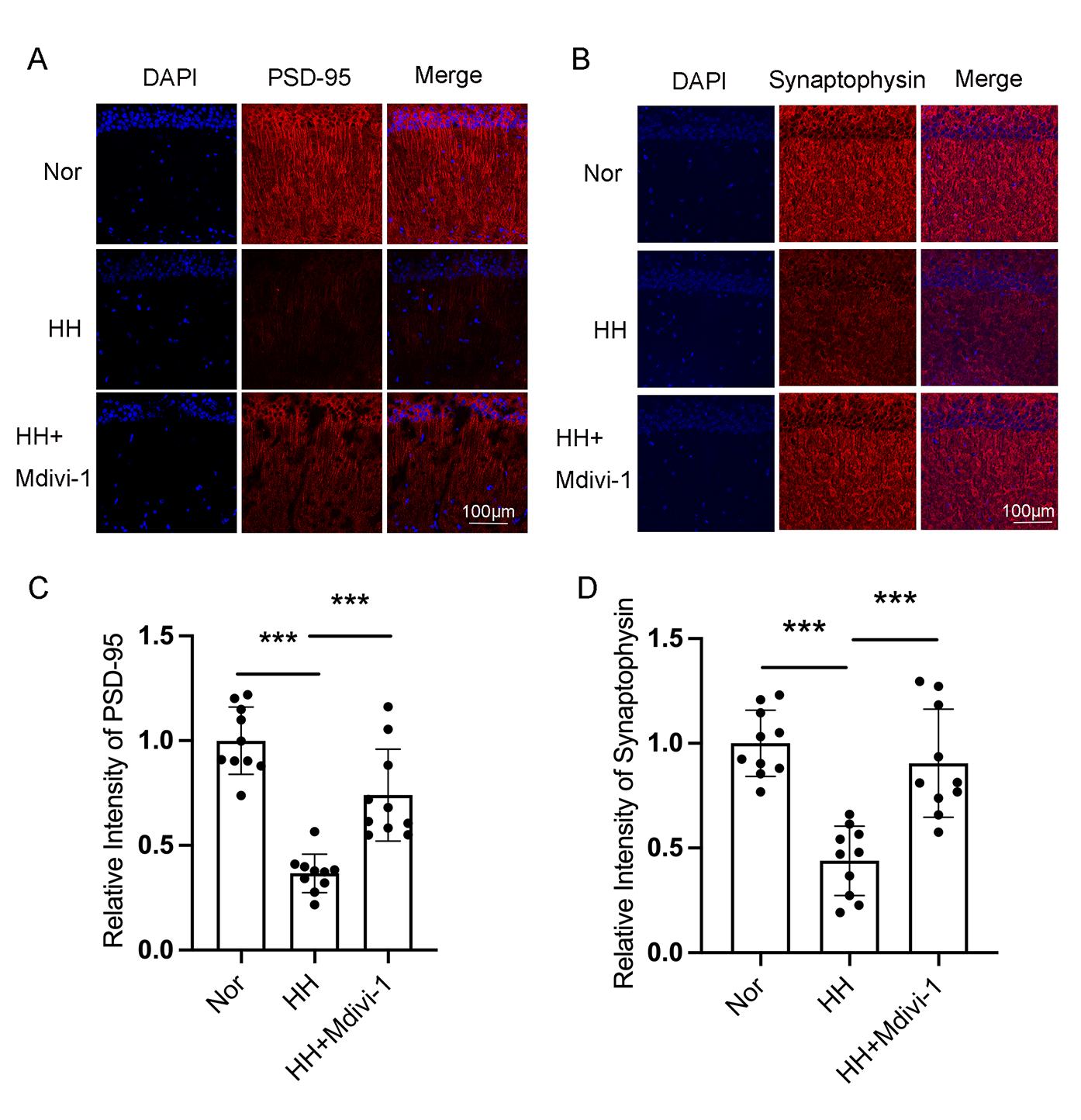

Supplement: Supplementary file 1 — Figure S1. Mdivi‐1 attenuated the reduction in the number of synapses in mice brains induced by long‐term, intermittent simulated high‐altitude exposure. Mice were pretreated with Mdivi‐1 and then exposed to HH 3 times as described in Figure 1A. (A, B) The levels of PSD‐95 and Synaptophysin were observed by immunofluorescence, respectively. Scale bar = 100 μm. (C, D) Quantitative analysis of the intensity of PSD‐95 and Synaptophysin was performed in panels (A, B), respectively. Data were expressed as means ± SD (n = 10). ***p < 0.001. [file CNS-31-e70473-s001.tif]
